# Chronic Abscess of the Cheek, from the Irritation of a Carious Tooth
*Mr. Fletcher's Medico-Chirurgical Illustrations.


**Published:** 1831

**Authors:** 


### Chronic Abscess of the Cheek, from the Irritation of a Carious Tooth.*

The swelling from this cause, which often simulates a distinct tumour, is of a pale and purplish tint, little elevated, giving something of the appearance of a strumous abscess, though it feels firmer, and the skin looks thicker. Mr. Fletcher has known this swelling cut out from the cheek as a tumour otherwise irremoveable; he has also known it opened, treated with caustic, and in fact in all sorts of ways but the right. On removing the carious tooth, with its fang or fangs, the cause of the disease, the tumour gradually disappears without further treatment. On opening the tumour we find a small quantity of unhealthy pus, mixed sometimes with a slight fungous growth, and the fangs of the tooth will have attached to them a small fungous growth of similar character. Mr. F. has never seen this chronic tumour opposite to any other teeth than the first molares of the lower jaw. The history of the disease appears to be this. The carious fang excites some inflammation in the socket, which generally suppurates, when the pressure of the matter induces the ulcerative process outwardly, for its escape. During this process the contiguous portion of the cheek sympathises slowly with the irritation, inflames, and gives rise to the chronic abscess in question. The effect on the cheek may take place from the irritation of the diseased tooth, before the inflammation in the socket has advanced to suppuration; or this last may not even happen at all.

* Mr. Fletcher's Medico-Chirurgical Illustrations.

